# Food Safety, Health Management, and Biosecurity Characteristics of Poultry Farms in Arusha City, Northern Tanzania, Along a Gradient of Intensification

**DOI:** 10.24248/EAHRJ-D-18-00034

**Published:** 2018-11-23

**Authors:** Emmanuel Sindiyo, Ruth Maganga, Kate M Thomas, Jackie Benschop, Emmanuel Swai, Gabriel Shirima, Ruth N Zadoks

**Affiliations:** a School of Life Sciences and Bio-Engineering, Nelson Mandela African Institution of Science and Technology, Arusha, Tanzania; b Institute of Biodiversity, Animal Health & Comparative Medicine, College of Medical, Veterinary and Life Sciences, University of Glasgow, Glasgow, UK; c Department of Preventive and Social Medicine, Dunedin School of Medicine, University of Otago, Dunedin, New Zealand; d ^*m*^EpiLab, School of Veterinary Science, Massey University, Palmerston North, New Zealand; e Ministry of Livestock and Fisheries Development, Dar es Salaam, Tanzania

## Abstract

**Background:**

With the growth, urbanisation, and changing consumption patterns of Tanzania's human population, new livestock production systems are emerging. Intensification of poultry production may result in opportunities and threats for food safety, such as improved awareness of biosecurity or increasing prevalence of foodborne pathogens including nontyphoidal *Salmonella* or *Campylobacter* spp. We conducted a semiquantitative analysis of poultry production systems in northern Tanzania, with emphasis on biosecurity, health management practices, and prevalence of foodborne pathogens, to gain insight into potential associations between intensification and food safety.

**Methods:**

Interviews were conducted with managers of 40 poultry farms, with equal representation of 4 production systems (extensive, semi-intensive, or intensive production with indigenous chickens, and broiler farming). Per farm, up to 10 birds (total, 386) were tested for cloacal shedding of nontyphoidal *Salmonella*, with a subset of farms tested for *Campylobacter*. Data were analysed using univariate statistics, and results were discussed during feedback workshops with participating farmers and extension officers.

**Results:**

Clear differences existed between farm types with regard to implementation of biosecurity and health management practices and use of extension services. By contrast, prevalence of foodborne pathogens (6 of 40 farms or 15% for nontyphoidal *Salmonella* and 13 of 26 farms or 50% for *Campylobacter* spp.) was not farm-type specific, indicating that it is driven by other factors. Across farming systems, knowledge and awareness of the presence of antimicrobials in poultry feed and the need to abide by post-treatment withdrawal times were limited, as was access to impartial professional advice regarding treatment.

**Conclusion:**

Different control measures may be needed to protect poultry health compared to public health, and improvements in information provision may be needed for both.

## INTRODUCTION

Urbanisation in Tanzania increased from 5.7% in 1967 to 29.1% in 2012, and urban areas absorbed 12 million people out of a total growth of 31.6 million over that period.^1^ Urbanisation is associated with a growth in mean wealth – the value of assets owned – per capita, which increased from US $250 in 2004 to US $480 in 2012.^2^ Urbanisation and wealth drive chicken meat consumption, which is skewed towards medium-to high-income populations in urban areas.^3^ Tanzania has an estimated population of more than 43 million chickens.^4^ Considering demographic developments in the human population, an increase in poultry production can be anticipated. Indeed, in urban areas, such as Arusha Urban District, traditional extensive backyard poultry farming for home consumption is increasingly complemented by semi-intensive and intensive farming systems, with sales of poultry meat to individual customers, retailers, and caterers. While the majority of chickens – estimated at 96% of the population – belong to indigenous breeds, intensive production of exotic broilers is increasingly widespread, as evidenced by the presence of farms with such breeds in most wards of Arusha District.^4^ Poultry make a significant contribution to human nutrition and provide a source of income for farmers to support their livelihoods.^5,6^ Poultry also have important social roles in ceremonies and rituals or as gifts.^6^

While poultry make major contributions to the economy and social life in Tanzania, they may also expose farmers, consumers, and the environment to causative agents of zoonotic infections and foodborne diseases, notably through direct contact with birds or their excreta, or through handling or consumption of poultry meat or eggs contaminated with bacteria, such as *Campylobacter* spp. or *Salmonella* spp. Both genera can be carried asymptomatically by healthy birds, so it may not be obvious that a microbiological hazard is present.^7,8^ Globally, *Campylobacter* spp. and nontyphoidal *Salmonella* spp. are among the most important foodborne zoonotic pathogens. *Campylobacter* is the most common bacterial cause of foodborne illness, and *Salmonella enterica* is a major bacterial cause of mortality, associated with an estimated 230,000 deaths per year.^9^ The burden of *Salmonella* is particularly high in sub-Saharan Africa, and it is an important cause of febrile illness among hospitalised children in rural and urban Tanzania.^9–12^ Healthy poultry and poultry products are considered potential sources of both *Campylobacter* and *Salmonella*. A high prevalence of the bacteria in live birds increases the risk of contamination of chicken carcasses.^13–15^ Specific risk factors for *Salmonella* prevalence in poultry flocks have been studied in production systems in high-income countries, but information on risk factors in traditional or emerging African production systems is scarce.^16^ The risk of *Salmonella* contamination can be high in intensive poultry production, particularly if biosecurity is poor.^17^ However, intensification does not necessarily increase the prevalence of *Salmonella* or *Campylobacter*. For example, both the rise and subsequent fall of *Salmonella enterica* serovar Enteritidis were associated with intensive poultry production in the United Kingdom (UK), and *Campylobacter* prevalence was higher in extensively managed indigenous chickens than in intensively managed broilers in the Morogoro Region and Eastern Zone of Tanzania.^8,15,18^ Thus, the emergence of new poultry production systems may bring new risks of foodborne disease as well as new opportunities for human or animal disease prevention.

Our aim was to gain insights into the association between emerging poultry production systems and health risks and opportunities, and to explore suitable routes for dissemination of extension information to promote poultry health and public health. To this end, we conducted a semi-quantitative analysis of poultry production systems at different levels of intensification, with emphasis on the prevalence of foodborne pathogens, biosecurity, health management practices, and sources of medicines and health information.

## METHODS

### Study Area and Study Farms

**FIGURE 1.**
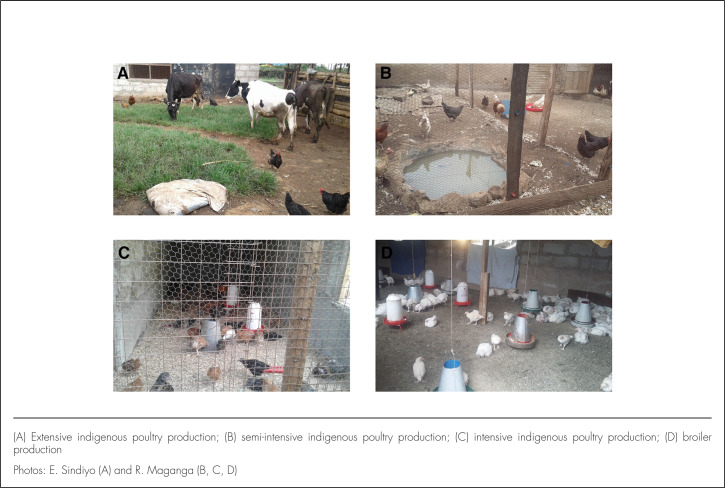
Examples of Poultry Production Systems at Different Levels of Intensification

The study was conducted in Arusha Urban District, which is among the 7 districts of Arusha Region in northern Tanzania. The district is a hub for tourism and is undergoing rapid economic expansion and urbanisation. It is located between longitudes 34.5° E and 38° E and latitudes 2° S and 6° S and is divided into administrative units called wards. Over the course of the study, the number of wards changed from 25 to 19 as a result of amalgamation. Farming systems for poultry in this area include intensive broiler production, intensive indigenous chicken production, semi-intensive indigenous chicken production, and extensive or free-range indigenous chicken production. The major difference between the various indigenous poultry management systems is in the housing system, which can be described as permanently housed, partly housed, or not housed (**Figure 1**). Furthermore, the indigenous farming systems differ in their use of commercial feed, mixed commercial and home-made feed, and scavenging for poultry nutrition, respectively. Broilers differ from indigenous chickens in that they are bred and raised specifically for meat production. Broiler production is more intensive than production of meat or eggs with indigenous chickens.

(A) Extensive indigenous poultry production; (B) semi-intensive indigenous poultry production; (C) intensive indigenous poultry production; (D) broiler production

Photos: E. Sindiyo (A) and R. Maganga (B, C, D)

Because the aim of the study was to obtain information on poultry management and prevalence of foodborne pathogens across poultry production studies, only wards with all 4 systems were eligible for inclusion in the study. After permission for research in the district had been granted by the Arusha District executive director, poultry subject matter specialists within the district's agricultural extension service identified 20 of 25 wards as having all production systems. Out of 20 wards, 10 were selected at random by drawing names from a box: Elerai, Engutoto, Kimandolu, Lemara, Moshono, Muriet, Sinon, Sombetini, Terat, and Themi. For every selected ward, extension officers were asked to produce a list of poultry farmers, stratified by poultry farming system. From this sampling frame, 1 farmer was randomly selected per production system per ward by drawing names, written on pieces of paper, from a box.

### Data and Sample Collection

To collect information on farming households and poultry management, including husbandry and animal health-related practices, a semi-structured questionnaire was developed in English. During use, the investigator translated the questions into Kiswahili. Pilot testing of the questionnaire was conducted with 1 household per farming system in Sokon Ward to ascertain clarity of the questions and the amount of time needed for completion of all questions. Farmers were contacted by telephone to arrange a time for interview, and the questionnaire was administered to 40 farmers in person by the first author after obtaining the farmer's verbal consent in front of a witness. Questionnaires were completed before sample collection for 2 reasons: to explain in advance to the farmer how the sampling would take place and to allow for sampling of all farms within a ward (1 each for extensive, semi-intensive, intensive, and broiler production systems) in a single day. The latter was deemed important to avoid temporal bias in culture results from different production systems and would not have been possible if questionnaires also had to be conducted on the same day. Geographical positioning system data were collected for each household using an eTrex 10 device (Garmin, Southampton, UK).

Sampling of chickens and their environment was conducted once a week to allow sufficient time for sample processing between sample collection days. Chickens were handled gently to avoid injury, in compliance with the United Republic of Tanzania's Animal Welfare Act no. 19, part V, section 40-48, 2008.^19^ Cloacal swabs were collected by inserting the entire tip of a swab into the cloaca of a chicken and applying gentle pressure against the mucosal surface while swabbing in a circular motion. Each chicken was swabbed twice, once with a plain Amies swab and once with an Amies charcoal swab (Thermo Fisher Scientific, Newport, UK). Swabs were removed gently and immediately inserted into the respective Amies tubes, and then labelled and stored in cool boxes with ice packs before being transported to the laboratory for analysis within 5 hours – the time between the first sample collection and arrival at the laboratory. Environmental samples were collected by using 1 pair of boot cover swabs (BTSW-001 DRY Sterile Boot Cover Swab for Sampling Poultry Housing, Solar Biologicals Inc., Newark, NJ, USA) per farm and walking along the diagonals of the chicken house or yard. Dry boot cover swabs were used rather than premoistened swabs to avoid bacterial growth prior to use, which was deemed a risk under Tanzanian temperature and moisture conditions. Boot socks were worn over boot covers (Fearing Disposable Boot Covers, Smiths Animal Health, Ashbourne, UK) as per the directions of the boot sock manufacturer. Used boot cover swabs were stored in stomacher bags and transported to the laboratory together with the swabs. After collection of environmental samples and cloacal swabs on a farm and before visiting the next farm, all disposable personal protective equipment was changed, and hands and boots were disinfected using 70% ethanol.

### Sample Processing

Samples were processed in the bespoke Zoonoses Unit of the Biotechnology Laboratory at the Kilimanjaro Clinical Research Institute in Moshi, Tanzania.^20^ Culture methods were based on recommendations from the Food and Drug Administration's Bacteriological Analytical Manual for *Campylobacter* and *Salmonella*.^21,22^ The *Campylobacter* culture was initiated on the day of sample collection. All reagents were obtained from Oxoid (Basingstoke, UK) unless stated otherwise. Amies charcoal swabs were removed from transport containers and tips removed aseptically by cutting them off into a plastic universal tube containing 20 ml Bolton broth supplemented with 5% horse blood (TCS Biosciences, Botoph Claydon, Buckingham, UK) and selective supplement SR0208E, vortexed aseptically for 10 seconds and placed into a microaerophilic jar with CampyGen sachets. Samples were incubated at 37±2°C for at least 4 hours before being moved to 42±2°C for a further 42 to 46 hours, and then plated onto modified charcoal cefoperazone deoxycholate agar plates and incubated at 42±2°C in a micro-aerophilic jar with CampyGen sachets for 48 hours. Plates were examined for typical *Campylobacter* colonies. Suspect colonies were subcultured onto Columbia blood agar, incubated microaerophilically at 42±2°C for 48 hours, and subjected to oxidase and catalase testing and Gram staining for confirmation.

Samples for *Salmonella* detection were stored overnight in a refrigerator between 2°C and 8°C. Tips were aseptically removed from the plain Amies swabs the next day, placed in 20 ml buffered peptone water, vortexed for 10 seconds, and incubated at 37±2°C for 18 to 20 hours. A small volume (0.1 ml) of the enriched buffered peptone water was then transferred into 10 ml of Rappaport-Vassiliadis soya peptone broth and incubated at 42±2°C for 24 hours. One loopful (10 ll) of overnight culture was transferred onto xylose ly-sine deoxycholate agar with 5 μg/ml novobiocin (Sigma-Aldrich, St. Louis, MO, USA) and streaked for isolation. At least 2 typical *Salmonella* colonies per plate were streaked onto MacConkey agar and incubated overnight at 37±2°C. Lactose-fermenting colonies (those with a pink appearance) were discarded, and nonlactose-fermenting colonies were individually transferred into 5 ml of tryptone broth and incubated at 37±2°C for 4 to 24 hours. Growth from the broth was inoculated onto MacConkey agar to check for purity, then stabbed into lysine iron agar slopes and triple-sugar iron slopes and incubated overnight at 37±2°C to assess phenotype. Kovacks' indole reagent (Merck KGaA, Darmstadt, Germany) was added to the incubated tryptone broth to test for indole production. Presumptive identification of *Salmonella* isolates was based on negative indole test results, alkaline slant and butt (purple colour) in lysine iron agar, and red slope with yellow butt and gas production on triple-sugar iron slopes. Identity was confirmed by testing with poly-H and poly-O agglutination tests (Statens Serum Institut, Copenhagen, Denmark) and Microbact 12A test strips, following the manufacturers' instructions.

### Data Analysis

Data were stored and checked for missing values and outliers in Microsoft Excel (Microsoft, Seattle, WA, USA), with additional processing using Excel for visual analysis and Statistix 10 (Analytical Software, La Jolla, CA, USA) for quantitative analysis. To test for an association between farm type and categorical variables (eg, biosecurity characteristics or health management), chi-square (X^2^) analysis was used. Unless stated otherwise, there were 3 degrees of freedom for X^2^ testing, based on analysis of binary variables across 4 farm types. Nonparametric Kruskal–Wallis ANOVA was used for continuous variables. Statistical significance was declared at *P*<.05. To generate a map of the study area showing the production system and culture results for each farm, QGIS software, version 2.18.3 (https://qgis.org/en/site/) was used.

### Feedback Sessions

Two-day feedback sessions were held with poultry keepers and extension officers in Engutoto Ward and at the Arusha Veterinary Investigation Centre, respectively. The aim of the feedback sessions was to share results from the study, create awareness of biosecurity and health management among poultry keepers and extension officers, and to collect their views on current service provision and needs. After initial introductions and presentation of the results, participatory approaches were used, including group discussions guided by questions and opportunities for participants to present their views. Group discussions were facilitated by the first author, who also arranged the farm visits – with help from the extension officers – and administered the questionnaires to the farmers. The first author was selected for this role because of his knowledge of the subject matter, local conditions, and terminology, as well as the rapport that he had developed with the participants through the project; this facilitated informed and open dialogue.

### Ethical Approvals

Ethical approval for this work was provided by the National Institute for Medical Research (NIMR/HQ/R.8a/Vol.IX/2028) and the Kilimanjaro Christian Medical Centre (Research Ethical Certificate No. 832), as part of the Zoonoses and Emerging Livestock Systems project. Approval to conduct the interviews of human subjects was granted by the University of Glasgow College of Medical, Veterinary and Life Science's Ethics Committee (200140183), and poultry sampling was approved by the University of Glasgow School of Veterinary Medicine Research Ethics Committee (Ref. 56a/16). A letter of approval was provided by the Municipal Council of Arusha Urban District, where the research took place.

All interviewees provided informed consent before participating in the study. Consent was given verbally in the presence of extension officers rather than in writing to prevent exclusion of participants based on literacy.^20^ Details that might disclose the identity of participants in the study are not shown.

## RESULTS

### Prevalence of Foodborne Pathogens

**FIGURE 2.**
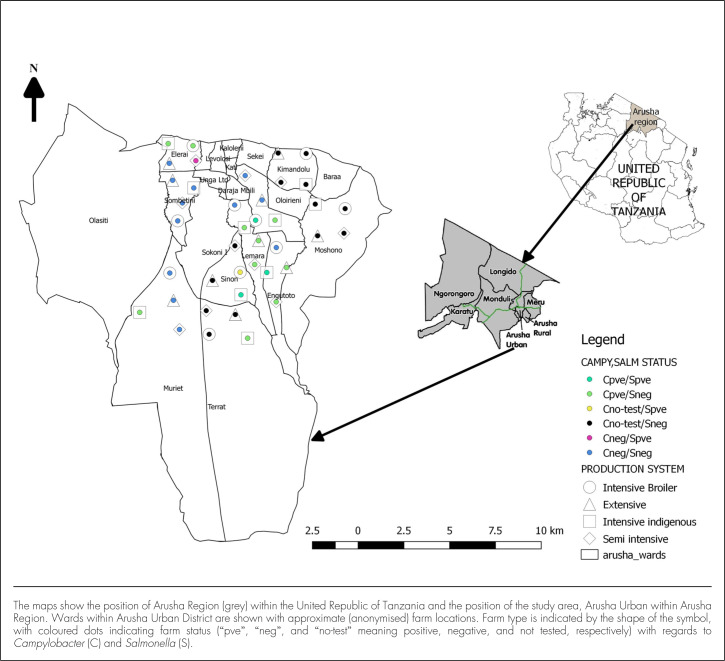
Map of Study Area Showing *Campylobacter* and *Salmonella* Status by Farm Type and Location

Visits and interviews were conducted at 40 farms, divided over 4 production systems and 10 wards, with 1 farm per production system per ward. Out of a target number of 400 birds, 386 were swabbed: 99 from broiler flocks (9 farms with 10 birds, 1 farm with 9 birds), 99 from intensive flocks (9 farms with 10 birds, 1 farm with 9 birds), 98 from semi-intensive flocks (8 farms with 10 birds, 2 farms with 9 birds), and 90 from extensive flocks (8 farms with 10 birds, 2 farms with 5 birds). Environmental samples were collected from all farms. Six (15%) of 40 farms and 8 (2.1%) of 386 birds tested positive for *Salmonella*. Increased farm intensification was associated with nonsignificant increases in the numbers of positive farms and birds (**Table 1**; X^2^=2.3, *P*=.51 at farm level; X^2^=4.6, *P*=.20 at bird level). Due to logistic issues, samples from 26 farms only were tested for *Campylobacter*, of which 13 (50%) were positive. Animal-level prevalence of *Campylobacter* (23 of 255 birds, 9.0%) was higher than for *Salmonella* but without an obvious association with farm intensity (**Table 1**). Joint occurrence of *Salmonella* and *Campylobacter* was detected on 3 farms, as would be expected by chance under the assumption of independent occurrence of the 2 bacterial genera. The distribution of farms in the study region, including farm type and farm status, with regards to *Salmonella* and *Campylobacter*, is shown in **Figure 2**.

**TABLE 1.** Prevalence of *Campylobacter* spp. and Nontyphoidal *Salmonella* in Tanzanian Poultry Farms Across a Gradient of Intensification

Pathogen	Farm Type	Farm Level Positive/Tested (%)^a^	Bird Level Positive/Tested (%)	Boot Socks Positive/Tested (%)
*Campylobacter* spp.	Extensive	2/6	5/55 (9.1)	NA
	Semi-intensive	3/6	7/60 (6.7)	NA
	Intensive	7/8	12/80 (15.0)	NA
	Broiler	1/6	3/60 (5.0)	NA
	All	13/26 (50)	27/255 (10.6)	NA
Nontyphoidal *Salmonella*	Extensive	0/10	0/90 (0.0)	0/10
	Semi-intensive	1/10	1/98 (1.0)	1/10
	Intensive	2/10	3/99 (3.0)	2/10
	Broiler	2/10	4/99 (4.0)	2/10
	All	5/40 (12.5)^b^	8/386 (2.1)	5/40 (12.5)^b^

a Percentage only calculated for denominator values greater than 25.

b In total, 6 of 40 farms were positive for *Salmonella*: 1 semi-intensive farm, 2 intensive farms, and 3 broiler farms (1 farm demonstrated positivity via cloacal swabs only, 1 farm via boot socks only, and 4 farms via both).

Abbreviations: NA, not applicable; spp., several species.

The maps show the position of Arusha Region (grey) within the United Republic of Tanzania and the position of the study area, Arusha Urban within Arusha Region. Wards within Arusha Urban District are shown with approximate (anonymised) farm locations. Farm type is indicated by the shape of the symbol, with coloured dots indicating farm status (“pve”, “neg”, and “no-test” meaning positive, negative, and not tested, respectively) with regards to *Campylobacter* (C) and *Salmonella* (S).

### Farmer Demographics

Poultry management was generally the responsibility of women, with a mean of 7 of 10 farms per production system managed by a woman (range, 6 to 8). Only 2 interviewees identified chicken production as their main occupation. Other sources of income included crop production, formal or informal business, and civil service. The majority (n=32, 80.0%) of people responsible for chicken management were over 40 years of age. Of those under 40 years of age, half managed semi-intensive farms, and only 1 was younger than 30. A wide range of education levels was reported, from primary school education (standard 7, equivalent to 7 years of primary education up to age 13), via ordinary and advanced secondary education (form 4 and form 6, respectively), to postsecondary and adult education. Broiler farming was the only sector where none of the respondents reported university-level education, although differences between sectors were not significant. Half of the participants on extensive and semi-intensive farms reported to have skills in poultry production, as did the majority of participants on intensive (8 of 10) and broiler (9 of 10) farms.

### Husbandry

On extensive farms, 13 to 75 birds (mean, 39; median, 34) were housed in a single chicken house. On semi-intensive farms, 35 to 105 birds (mean, 57; median, 49) were housed in 1 to 4 houses (median, 1). On intensive farms, 15 to 700 birds (mean, 199; median, 113) were distributed over 1 to 4 houses (median, 2). Finally, on broiler farms, there were 200 to 1,500 birds (mean, 715; median, 600) across 1 to 3 houses (median, 3). The number of birds was significantly higher on broiler farms than on extensive or semi-intensive farms, whereas the number of houses per farm was significantly higher on broiler and intensive farms than on extensive farms (Kruskal–Wallis 1-way ANOVA with post-hoc Dunn's pairwise comparison, *P*<.001 for both analyses). Bedding use reflected intensification of the production system, with litter used on 10, 4, 3, and 1 broiler, intensive, semi-intensive, and extensive farms, respectively (X^2^=18.2, *P*<.001). Chickens were fed tap water in 5 to 8 farms per farm type, and only extensive farmers used river water. Commercial feed was used on all broiler farms, and homemade feed was used on 9 of 10 intensive farms. Semi-intensive farms used a variety of feed sources, and birds scavenged for food on all extensive farms. All farmers fed their chickens minerals, multivitamins, or both. All producers had purchased their birds, except for 2 extensive producers and 1 semi-intensive producer, who received chickens as gifts.

### Biosecurity

**FIGURE 3.**
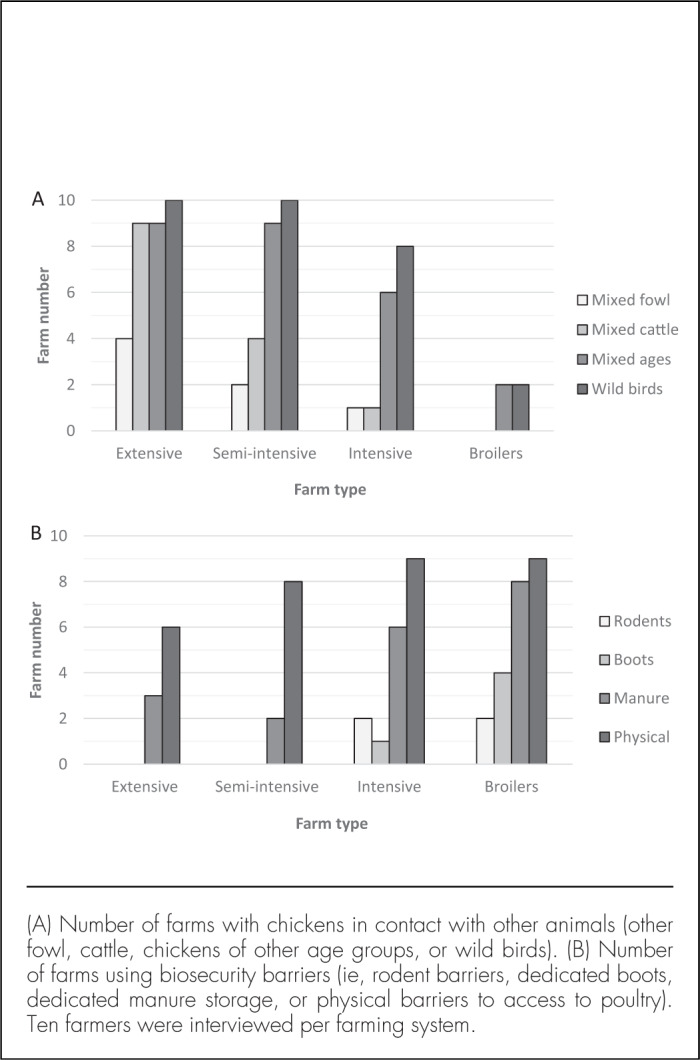
Association Between Farm Intensification and Biological and Physical Biosecurity in Poultry Production Systems in Arusha Urban District, Northern Tanzania

Biosecurity improved as farm intensification increased (**Figure 1** and **Figure 3**). Mixing of birds of different age groups was common on extensive and semi-intensive farms but not on broiler farms (X^2^=14.5, *P*=.002). With intensification, the number of farms where chickens mixed with other types of fowl decreased (X^2^=6.1, *P*=.11), as did the number of farms where chickens were in contact with ruminant species (cattle, n=14, X^2^=21.5, *P*<.001; goats, n=8, X^2^=11.3, *P*=.01; sheep n=6, X^2^=7.1, *P*=.07). Other types of fowl included ducks, geese, and turkeys on n=7, 3, and 3 farms, respectively. Contact with wild birds was common on most farms other than broiler farms (X^2^=22.9, *P*<.001), and all farms reported contact of chickens with rodents, except for a single broiler farm. Contact was also reported with dogs, cats, donkeys, and bats, but not with pigs. The presence of layer hens was reported on half of the extensive farms and most of the semi-intensive and intensive farms but not on broiler farms. All broiler farms practised the all-in, all-out system, but none of the other farms did. Sick chickens were generally not removed from farms, regardless of farm type, although some were sacrificed (on 2 broiler farms and 1 intensive farm), sold (2 intensive farms), or slaughtered for home consumption (on 1 broiler, 1, intensive, 3 semi-intensive, and 4 extensive farms). Physical barriers limiting access to chickens, separate manure storage, dedicated boots, and rodent barriers were generally more common at the higher levels of intensification (**Figure 3B**). The association with farm type was significant for manure storage (X^2^=9.1, *P*=.028) and use of dedicated boots (X^2^=9.8, *P*=.020), but not for the other barriers, nor for the use of food baths, which was limited to a single broiler farm.

(A) Number of farms with chickens in contact with other animals (other fowl, cattle, chickens of other age groups, or wild birds). (B) Number of farms using biosecurity barriers (ie, rodent barriers, dedicated boots, dedicated manure storage, or physical barriers to access to poultry). Ten farmers were interviewed per farming system.

### Health Management

Vaccines to prevent viral diseases were commonly used, with half of the farmers using a vaccine against Newcastle disease (**Table 2**). Vaccination against Newcastle disease was significantly more common on extensive and semi-intensive farms, and pox vaccination was more common on intensive and broiler farms. Half of the farmers reported use of anti-helminthics, with a nonsignificant association between anti-helminthic administration and farm intensification (**Table 2**). Antimicrobial use was reported by a clear majority (n=38, 95.0%) of farmers, whereas traditional herbs were predominantly used by extensive farmers. Routine use of antimicrobials was significantly more common on broiler farms than other farm types (*P*=.002), where antimicrobials were reported to be used occasionally or only when birds were sick. With a few exceptions (1 each among extensive and semi-intensive farms, and 2 among intensive farms), treatments were administered to the entire flock rather than to individual sick birds. The choice of drugs was mostly based on advice from drug sellers, with a minority of farmers primarily relying on veterinary advice or personal experience. Only 1 semi-intensive farmer reported consulting an extension officer before treatment. Few farmers were aware that poultry feed might contain antimicrobials. Across farming systems, almost half of all farmers said that they were aware of the impact of antimicrobial residue on human health and the existence of withdrawal times after antimicrobial use, but only a quarter abided by rules around withdrawal times (**Table 2**).

**TABLE 2.** Use and Knowledge of Vaccines and Drugs on Tanzanian Poultry Farms Across a Gradient of Intensification

	Farm Type					Statistics	
Topic	Total n	Broiler n	Intensive n	Semi-intensive n	Extensive n	Chi-square	*P* Value
Vaccines							
Gumboro	1	0	1	0	0	3.1	.38
Newcastle disease	20	0	5	7	8	15.2	.002
Pox	13	6	5	2	0	10.4	<.02
Drugs							
Antihelminthics	20	7	6	4	3	4.0	.26
Antimicrobials							
Routinely	10	7	1	1	1	14.4	.002
Occasionally	12	2	5	4	1	4.8	.19
When birds are sick	16	1	4	5	6	5.8	.12
Traditional herbs	8	1	2	0	5	8.8	.03
Drug choice based on							
Personal experience	9	4	2	3	0	5.0	.17
Drug seller's advice	21	4	4	5	8	4.3	.23
Veterinary advice	9	2	4	2	1	2.5	.48
Knowledge of							
Antimicrobials in poultry feed	4	2	1	1	0	2.2	.53
Residue impact on people	17	5	4	4	4	0.3	.99
Withdrawal time							
Aware	17	7	5	3	3	4.4	.22
Abides	9	3	3	1	2	1.6	.66

### Extension and Training

All farmers expressed the need to receive information on poultry keeping. Most farmers were members of 1 or 2 professional groups, including farmer field schools or poultry associations, but only a minority considered this useful. Most farmers – particularly broiler farmers – relied on input suppliers for extension services. Some farmers – particularly those on extensive farms – relied on government extension officers for extension services. Information on poultry farming was mostly obtained from colleagues and occasionally from farmer field schools, input suppliers, or social media. None of the associations between information source and farm type were significant (**Table 3**). Farmers' own resources were the most common sources of funding for training on all but extensive farms, where the government was the most common source of funding for access to information (data not shown). Nongovernmental organisations occasionally funded access to information, but they were never cited as the main source of information.

**TABLE 3.** Engagement of Poultry Farmers With Farmers Groups, Extension and Information Providers, and Vaccine Suppliers Across a Gradient of Farm Intensification in Northern Tanzania

	Farm Type					Statistics^a^	
Topic	Total n	Broiler n	Intensive n	Semi-intensive n	Extensive n	Chi-square	*P* Value
Farm group membership^b^	32	6	10	8	8	5.0	.17
Farmer field school	25	8	5	6	6	2.0	.57
Poultry association	15	2	5	4	4	1.3	.72
Useful	10	3	2	1	4	2.7	.46
Main extension provider							
Government	8	0	1	3	4	6.3	.10
Input supplier	21	8	5	4	4	4.3	.23
Nongovernmental organisation	1	0	1	0	0	3.1	.38
Information sources							
Farmer field school	8	1	1	2	1	0.7	.88
Input supplier	5	1	1	2	1	0.7	.88
Social media	5	1	1	3	3	2.5	.48
Colleagues	22	7	7	3	5	4.4	.22
Vaccine provider							
Government extension	1	0	0	1	0	3.1	.38
Input supplier	38	10	10	9	9	2.1	.55

a *P* values indicate significance of an association between farm type and engagement (yes/no) based on chi-square analysis.

b Ten farmers were interviewed per farm type, and numbers indicate the farmers using the specified membership or service. Some farmers did not use any of the service providers listed, so numbers may not add up to 10 per farm type.

Issues impacting the use of extension services by farmers were identified by farmers and extension officers in separate feedback sessions. The 2 major issues identified by both groups were timeliness of the extension officers' responses to requests from farmers and the fact that extension officers provide advice without being able to offer treatment or vaccination. Timeliness of service provision was affected by a lack of available transport and by competing demands on the extension officers' time, while the quality of the service that could be offered was affected by a lack of mentoring, extension kits, and medicines. An additional issue was the lack of appropriate introductions of extension officers to farmers by the relevant authorities. Private veterinarians and input suppliers can provide advice more quickly. Moreover, they have the ability to offer treatment products, although farmers recognised that they sometimes prescribe drugs that are available in their shop without due consideration of the suitability of the treatment. Suggestions for improvement largely revolved around related issues, including provision of transport and extension kits and changes to the chain of command for extension officers. In addition to health information, farmers desired information that could help them develop their business and access markets, as well as government involvement in inspections of hatcheries and parent stock. Feedback from research was particularly valued by extension officers and was summarised as follows in a vote of thanks on behalf of the group: “It is my first time in 25 years working experience to receive feedback from researchers, so we thank you very much and send our message to your sponsors and universities: We welcome you again”.

## DISCUSSION

*Campylobacter* and nontyphoidal *Salmonella* are important human pathogens in sub-Saharan Africa and may be transmitted through food of animal origin, including poultry products derived from healthy birds. Many foodborne human pathogens are commensals in the gastrointestinal tracts of animals, ie, bacteria that are carried without causing disease. Indeed, all *Salmonella* and *Campylobacter* isolates in the current study originated from clinically healthy birds. Small-scale outbreaks of foodborne disease due to contamination of human food with enteric commensals from animals have probably occurred throughout human history. They gained prominence in public health and scientific research in the latter part of the 20th century, when large outbreaks of salmonellosis and listeriosis in the United States and mortality due to *Escherichia coli* O157 stimulated public awareness and the development of the scientific discipline of food safety.^23–25^ Several major foodborne disease outbreaks in the United States and the United Kingdom occurred as a result of intensification and expansion of food production and distribution networks – processes that are currently taking place in much of sub-Saharan Africa.^24,25^

Traditional poultry keeping practices in Tanzania are changing as the country's poultry industry expands to meet the demands of a growing and increasingly urban consumer population. While intensification of food production is needed to provide food security, it must not come at the expense of food safety. Development and implementation of hazard analysis critical control point (HACCP) approaches across networks in the food industry may limit the risk of foodborne disease. For example, implementation of the Lion Code to control *Salmonella* Enteritidis in the British poultry industry has been followed by a significant decrease in human infections with this organism.^26,27^ In Africa, intensification of poultry production has been linked with increased prevalence of *Salmonella* and decreased prevalence of *Campylobacter*, but little is known about the association between farm management, biosecurity, and pathogen prevalence in relation to the emerging poultry systems in Tanzania.^8,17^ Moreover, it is largely unknown how farmers access information on these topics.

### Specific Risk Factors for the Presence of Foodborne Pathogens are Difficult to Identify

The prevalence of *Salmonella* in clinically healthy poultry was low in our study in Arusha, which is a positive outcome. A previous study of *Salmonella* in Tanzanian poultry also found a low prevalence, but that study focused on *Salmonella enter-ica* subspecies *enterica* serovar Gallinarum in layer hens.^28^ In our study, layers were not included, and serotyping of isolates was beyond the scope of this work, making it difficult to compare data across studies. A range of biosecurity measures were considered in our study, and many differed between farm types, including mixing of chickens with wild birds or ruminants. Although livestock, wild birds, and other wildlife may act as a source of *Salmonella* and introduce the organism into poultry flocks, we observed no association between farm types with different biosecurity levels and *Salmonella* prevalence.^7,29^

A lack of identifiable risk factors was also reported in a large study from Canada, where permanent locking of the poultry house was the only factor significantly associated with *Salmonella* prevalence.^16^ This risk factor was interpreted as a proxy for general biosecurity measures, but specific measures, such as boot washing, professional rodent control, or absence of contact with other host species were not significant.^16^ An alternative source of *Salmonella* exposure for chickens is poultry feed. A recent study on commercially produced chicken feeds from 3 feed mills in Dar es Salaam, Tanzania, showed that *Salmonella* prevalence ranged from 15% to 48% of feed bags, with significant differences between feed mills.^30^ This suggests that the “farm-to-fork” or “stable-to-table” concept should include poultry feed, as is the case with the British approach to *Salmonella* control.^26^ To determine the importance of feed as a source of *Salmonella* carriage in chickens or the importance of carriage in chickens as a source of human foodborne disease, strain typing of isolates from feed, chickens, and people would be required. In Burkina Faso, poultry, cattle, and pigs were shown to have similar levels of intestinal carriage of *Salmonella*, but only poultry isolates were genetically similar to those from humans, implicating poultry as the most likely source of human pathogens.^7^

Flock-level prevalence of *Campylobacter* carriage was 50% in our study, again without noticeable health impacts on the animals and without identification of specific risk factors, making it difficult to provide reasons and recommendations for *Campylobacter* control based on poultry health alone. Moreover, occurrence of *Salmonella* and *Campylobacter* was independent, suggesting that they are driven by different underlying processes and may require distinct control strategies. The fact that foodborne pathogens do not cause disease in animals poses a significant challenge because interventions that contribute to improved food safety and public health do not necessarily provide benefits to animal health. This is illustrated by the situation with *E. coli* O157:H7 in the United Kingdom, where vaccination of cattle would have major public health benefits but no animal health benefits, and uptake by farmers is low due to lack of economic incentives.^31^ Likewise, resource-constrained poultry producers in Tanzania may have low incentive to invest in control of food-borne pathogens that do not affect the health of their birds.

### Antimicrobial Use is Common in Poultry Production and May Pose a Risk to Public Health

In addition to the issues of food safety and food security, both of which should be considered One Health issues, a third One Health issue was identified through questionnaires: a lack of guidance and knowledge around the use of antimicrobials. Fewer than half of the farmers were aware of the existence of withdrawal times after antimicrobial use, and even fewer abided by the withdrawal guidelines. Considering global concerns about antimicrobial resistance (AMR), the observed lack of awareness and compliance with withdrawal times needs to be addressed. Awareness and compliance were more common among intensive and broiler farmers, hinting at potential benefits of intensification in terms of farmer education.

At the same time, broiler farmers were more likely to use antihelminthics and to use antimicrobials routinely. Broiler farmers were also more likely than other farmers to rely on input suppliers for extension services and on colleagues or personal experience for information and treatment decisions. Lack of independent, professional advice could contribute to frequent drug administration, which might contribute to higher selection pressure in favour of AMR, suggesting a potential hazard of farm intensification. The numbers in our study are small and associations are mostly nonsignificant, but the lack of unbiased professional input towards health management and treatment decisions may warrant more thorough socio-anthropological investigation of this issue. Tanzania's National Action Plan on AMR includes an analysis of strengths, opportunities, weaknesses, and threats and recognises that inadequate promotion of food safety along the chain and dispensing of antimicrobials by nonprofessionals are threats to AMR prevention.^32^

### Poultry Farmers and Extension Officers Agree on the Need for Improved Service Provision

The importance of communication and access to information and drugs were also raised in feedback workshops. The fact that extension officers offered advice on health management and disease prevention rather than products for disease treatment was seen as a major weakness of the service they provide. This has been a longstanding problem in preventive veterinary medicine throughout the world, and cycles of rise and fall in interest in preventive rather than curative approaches have been described in detail in the United Kingdom.^33^ Briefly, in times of need, urgency tends to take precedence over long-term consequences, and resources are diverted towards curative approaches. Use of resources for disease prevention is more likely in periods of relative wealth and calm. In Europe, differences still exist between production sectors, whereby preventive health management is now the standard on poultry farms, and cattle practice is often still largely responsive. Currently, only 20% of livestock farmers in Tanzania use livestock services.^34^ Policy priorities for improved livestock services were recently identified by means of a livestock field officer survey.^34^

The survey identified better transport, improved balance between administrative and technical duties, and supervision for livestock officers as policy priorities. These priorities were echoed in our feedback workshops. Additional priorities were regulation of fees charged by livestock officers – who may also act as private input suppliers – and better communication between central and local government staff on livestock-related policy.^34^ Our data suggest that improvement in communication is not only needed within the government-regulated livestock system but also between the government system and poultry producers, particularly producers in intensive systems. If trends in population growth and urbanisation continue, so will the intensification of poultry production. Considering that the average broiler flock in the study area was almost 20 times as large as the average extensive flock, a growing proportion of poultry meat will originate from broiler farms. Unbiased information on disease prevention and control, along with incentives to limit the use of antimicrobials and the risk of AMR, will become increasingly important as the intensification of poultry production continues.

### Limitations

This study had several limitations, such as the limited number of farms per production system and the narrow geographic focus on Arusha Urban District. However, all relevant levels of intensification were represented, and the information obtained from the study has yielded valuable insight into the complexity of managing poultry health and public health in an economically viable manner. Particularly, our results suggest that biosecurity measures, which farmers implement to protect poultry health, are not directly linked with the prevention of foodborne pathogens, and that different foodborne pathogens may have different drivers of prevalence. A much larger study would be needed to identify risk factors for all relevant poultry health and public health hazards, and it would need to be accompanied by economic studies to understand how to incentivise poultry keepers to take measures to prevent multiple hazards, including those that are not directly related to poultry health. A second limitation of this study is that *Salmonella* and *Campylobacter* isolates were not identified to strain level, and they were not compared with isolates of human origin. Thus, their potential contribution to the human disease burden was not demonstrated at the molecular level. Isolates have been archived at Kilimanjaro Clinical Research Institute so that molecular epidemiological investigations can be conducted at a future date.

## CONCLUSION

Population growth, urbanisation, and the associated emergence of intensified poultry production systems in Tanzania bring opportunities and risks for poultry farming, public health, and food safety. Based on our findings, biosecurity and awareness of antimicrobial residues is better on large, intensive farms than on small, extensive farms, implying that intensification may bring benefits for poultry health (reduced risk of disease introduction through better biosecurity) and for human health (reduced risk of antimicrobial residue in food for human consumption). In contrast to extensive producers, who receive advice from government extension officers, intensive producers tend to receive poultry health and treatment advice from private commercial suppliers who may have inherent conflicts of interest related to provision of information and products. This could contribute to overuse of antimicrobials and might constitute a risk to public health. Biosecurity measures were not linked to detection of *Salmonella* or *Campylobacter*, implying that farm management strategies to protect poultry health do not necessarily protect human health. Separate control strategies may need to be developed to limit the presence of foodborne pathogens. This is further complicated by the fact that occurrence of the 2 pathogens seems to be independent, suggesting that different transmission mechanism and control strategies are involved. For the sake of food security and public health, it seems important that the Tanzanian government develops ways to engage with its emerging poultry production system so that the potential benefits of intensification for biosecurity, food security, and food safety can be reaped without increasing the risk of overuse of antimicrobials.
